# Designing and Validating a Basketball Learning and Performance Assessment Instrument (BALPAI)

**DOI:** 10.3389/fpsyg.2019.01595

**Published:** 2019-07-10

**Authors:** Sergio J. Ibáñez, Sergio Martinez-Fernández, Sergio Gonzalez-Espinosa, Javier García-Rubio, Sebastián Feu

**Affiliations:** ^1^Faculty of Sport Science, University of Extremadura, Cáceres, Spain; ^2^Cáceres Club Baloncesto, Cáceres, Spain; ^3^Faculty of Education, University of Extremadura, Badajoz, Spain

**Keywords:** team sport, evaluation, validity, reliability, basketball, assessment

## Abstract

**Introduction:**

The assessment of learning in basketball in the PE class, and in training sessions of young players, requires valid, reliable, and trustworthy tools. The purpose of this research was to design and validate the Basketball Learning and Performance Assessment Instrument (BALPAI) that assesses simultaneously decision making, technical execution and efficacy. The play actions are codified using a categorical system, awarding a score for each category (1 = inadequate action; 2 = neutral action 3 = adequate action). An example of a summative procedure for assessing decision making in dribbling is: (1) Dribbling to a place where there is defensive pressure and there is a free teammate able to receive the pass; (2) Dribbling to a place where there is defensive pressure or a free teammate able to receive the pass; (3) Dribbling through a space where there is no defensive pressure and no free teammate able to receive the pass.

**Methods:**

A pilot study was performed following this procedure. A group of 13 experts participated in the assessment of the 33 elements (66 items) included in the BALPAI. Aiken’s V formula was used to analyze content validity, and internal consistency was calculated using Cronbach’s α. Inter-observer reliability was determined among three observers who used the BALPAI to record the play actions in a 3 × 3 basketball match (N = 45 possessions) and was calculated with the Multirater κfree, obtaining an almost perfect agreement with values between 0.84 and 1.

**Results:**

The BALPAI has very high internal consistency (0.969), Interobserver reliability was almost perfect (>0.84 in all items) and Aiken’s V coefficient (>0.71 in all items) attained a high value.

**Conclusion:**

The BALPAI proved to be a valid tool, with high internal consistency and reliability that makes it possible to perform a complete assessment of basketball in PE classes.

## Introduction

A mandatory prerequisite for making a valid and reliable qualitative analysis is the organization of the information by competent analysts. A systematic observational strategy (SOS) has to be planned (Knudson and Morrison, 2002) that contains all the relevant information about human movements. Therefore, the designing of instruments for the assessment of team sports using observation has become increasingly important as a research topic in the last few years. Between the 60s and the 80s, objective tests were the predominant method for assessing motor skills (Lopez-Pastor et al., 2013).

These tests presented certain limitations for being applied to the different playing skills in invasion sports as they did not include decision making (Oslin et al., 1998), and actual play during games (Bar-Eli and Raab, 2006). Later, test focused on the speed decision-makings occurs in real game (Thiffault, 1980), and evolve to measure the accuracy of these decisions (French and Thomas, 1987). Advances in the assessment of game performance behaviors help PE teachers to draw solid conclusions about their interventions during team sports teaching. The development of valid and reliable instruments will help not only teachers, but also other students for peer assessment in classroom settings (Memmert and Harvey, 2008). Moreover, linking what is being taught to what will be assessed, helps students to focus on what is important, making the teaching-learning cycle more congruent (Grehaigne et al., 2005). Accordingly, specific research should be undertaken in the evolution and development of observation tools that overcomes these limitations.

Thus, several instruments have been developed to assess play performance using systematic observation (Morgan et al., 2014), like the *Game Performance Assessment Instrument* (GPAI) (Oslin et al., 1998), or the *Team Sport Assessment Procedure* (TSAP) (Gréhaigne et al., 1997). These instruments represent a starting point for the observation of different team sports, and were designed to offer tools for PE teachers to assess their students. Based on these tools, new specific ones have been developed for different sports like soccer (García-López et al., 2013), handball (Tallir et al., 2003), futsal (Gonzalez-Villora and da Costa, 2015), or basketball (Tallir et al., 2007; Chen et al., 2013; Folle et al., 2014). These tools including the observation and assessment of a greater number of offensive actions with the ball (for example: shooting technique, dribbling technique, passing and catching, in and out 1 × 1, etc.) and without the ball (for example: jump stop and pivoting, 2 × 2 and 3 × 3 game play, etc.). In fact, off-the-ball actions are essential to be successful (Oslin et al., 1998), due the quantity of game play that occurs away from the ball.

In basketball, the tactical instrument proposed by Tallir et al. (2007) is more complete that the one proposed by Chen et al. (2013), as it analyses three different components for each play action (decision making, execution of the motor skill and efficacy). In each one of these components, appropriate and inappropriate actions are defined using a system of categories. Thus, it specifies what decisions are *correct* and *incorrect*, what technical actions are executed *correctly* and *incorrectly* and finally if the results of the actions are *successful* or *unsuccessful*. However, the limitation of these aforementioned instruments is that they only observe and assess offensive actions, not taking into account defensive ones.

The tools for observation and evaluation of the game must not only be reliable and valid, but must also be designed so they do not generate doubts in the observers, possessing high inter and intra-observer reliability. For this reason, it is necessary to complement the designs and validation of tools with real-time testing by observers or coders (Painczyk et al., 2018). These instruments present limitations as they do not take into account all the phases of the game (offense and defense), all the playing skills (with and without the ball) or the three components of play actions (decision making, technical execution and efficacy). In addition, as Knudson and Morrison (2002) stated, although their book is primarily based on technique, it is necessary to establish the level of the analysis. These instruments should assess the long- or short-term improvement of a motor skill, called *Learning* and *Performance.* Short-term changes in motor skills refer to performance, whereas long-term changes are called learning. Therefore, the objectives of the present study were (i) to design and validate an instrument for the specific and overall assessment of basketball, and (ii) to assess its inter-observer reliability. This instrument should make it possible to evaluate the learning of the basic concepts of the game that can be used in a 3 × 3, because it can be used to evaluate students in school (basic learning) and young basketball players who are beginning their training (basic performance).

## Materials and Methods

### Instrument

The measures tool, the Basketball Learning and Performance Assessment Instrument (BALPAI), e.g., the protocol to obtain the variables to be analyzed (see Supplementary Annex 1), includes a total of 11 play actions, 7 offensive play actions with and without the ball (Dribbling; Shooting; Passing; Receiving; Passing game; Occupying free spaces without the ball; Offensive rebound), and 4 on ball and off ball in defense (On ball defense; Off ball defense; Defensive help/defensive change; Defensive rebound). All these actions belong to the taxonomy of contents drawn up by Ibáñez (2002) for basketball training categories. Complex actions of the 3 × 3 game (pick and roll or pop, pin downs, hand-off, etc.) are not included, since the instrument has been designed to evaluate basic learning and performance. The inclusion of complex tactical actions, with multiple solutions, requires specific instruments, such as those designed to analyze the pick and roll (Gómez et al., 2015) or the inside pass (Courel-Ibáñez et al., 2016). The instrument assesses three differentiated components of play actions: decision making, technical execution and final efficacy. Each of these three components of the play action is codified according to its adequacy. Thus, each action is codified as: (i) Inadequate; (ii) Neutral; or (iii) Adequate. This codification proposal is different from the majority of existing instruments, as it is a development from two levels of assessment (appropriate/inappropriate; adequate/inadequate; successful/unsuccessful) to three, being similar to the one suggested by Folle et al. (2014), including an intermediate level of adequacy. The play actions are codified using a categorical system (Supplementary Annex 1), awarding a score for each category (1 = inadequate action; 2 = neutral action 3 = adequate action). Once all the play actions have been codified, the match participation and performance indicators are calculated for each player.

Two procedures were followed to establish the adequacy of the game action in each of the components of the instrument, [based on Chen et al. (2013) and Tallir et al. (2007)]: (i) the summative procedure, (ii) the levels procedure.

#### Summative Procedure

Two criteria were established to assess a game action. If the game action fulfills both established criteria, it is considered adequate; if it only fulfills one criterion it is considered neutral; and if it does not fulfill any criterion it is considered inadequate. An example of a summative procedure for assessing decision making in dribbling is: (1) Dribbling to a place where there is defensive pressure and there is a free teammate able to receive the pass; (2) Dribbling to a place where there is defensive pressure or a free teammate able to receive the pass; (3) Dribbling through a space where there is no defensive pressure and no free teammate able to receive the pass.

#### Levels Procedure

Three levels of adequacy were established for the action. Depending on how the action is observed, its level of adequacy is determined (inadequate, neutral or adequate). In addition, an example of the levels procedure for assessing final efficacy in shooting is: (1) The shot is blocked by a defender and/or does not touch the hoop or backboard; (2) The shot does not go through the hoop but touches the hoop or backboard; (3) The shot goes through the hoop.

After all the play actions have been codified, the indicators are calculated for participation in the game (PG), decision making (DM), technical execution (TE) and final efficacy (FE). The Performance Index (PI) in the game is calculated from these together with the decision-making performance index (DM-PI), the technical execution performance index (TE-PI), the final efficacy performance index (FE-PI); and the total performance index (Total-PI) (Table 1). These indices offer information on each of the analyzed dimensions and the game performance of the student or player.

**Table 1:** Calculation of indicators of participation and performance in the game.

	Decision making (DM)	Technical execution (TE)	Final Efficacy (FE)
	
PLAYER A	Sum of points for decision making (Pts DM)	Sum of points for technical execution (Pts TE)	Sum of points for final efficacy (Pts FE)
Participation in the game (PG)		PG = N° of total actions performed by player A	
Performance Index (PI)	PI-DM = Pts DM/PG	PI-TE = Pts TE/PG	PI-FE = Pts FE/PG
		Total PI = (PI-DM+PI-TE+PI-FE) /3	

### Research Design

This research represents an instrumental investigation as it involves the design and validation of an instrument for its subsequent application (Ato et al., 2013). For this reason, this section is organized in two studies.

#### Study 1: Design and Validation of an Instrument for the Specific and Overall Assessment of Basketball

##### Participants

In this study, the selection of the sample was intentional, as all the subjects chosen had to fulfill determined inclusion criteria to be able to be identified as experts. These criteria were based on their experience in making judgments, their reputation in the community, their availability and motivation for participating in the study, and their impartiality and inherent qualities, like self-confidence and adaptability (Skjong and Wentworth, 2001). Thus all the experts had to fulfill at least four of the following six criteria: (i) have a Ph.D. in Sports Sciences; (ii) be or have been a university lecturer; (iii) have the highest federative qualification in a team sport; (iv) have 10 years’ experience as a university lecturer; (v) have 10 years’ experience as a team sport coach in any category, and (vi) have published articles on the topic of team sports (Blomqvist et al., 2005; Villarejo et al., 2014; García-Martín et al., 2016; Ortega-Toro et al., 2019). All the experts were from the same country (Spain), and did not have a direct relationship with the research team. None of the experts received any gratification for participating in the project, their intervention being voluntary. Participation was requested from 25 experts who met the aforementioned requirements, and a response was received from 13 (52% participation). All experts signed written informed consents prior to the development of the study.

##### Measures

*Content validity*, which is the degree to which each item represents the content (Thomas et al., 2015). This variable was measured by expert judgment. The group of experts evaluated both the degree of pertinence of each item to the object of study (Adequacy), and the degree of accuracy and correctness in its explanation (Wording). Both concepts were evaluated with a Likert-type scale from 1 to 10. They were also asked for a general qualitative evaluation of each item to express possible alternatives when they deemed it necessary (Villarejo et al., 2014; García-Martín et al., 2016). The validity of the instrument was measured with Aiken’s V coefficient (Aiken, 1985).

*Internal* consistency or the reproducibility of the measure shows the internal reliability of the instrument A test cannot be valid if lacks of reliability. The have to be consistent to be trustworthy, results cannot depend on successive trials to achieve the same results (Thomas et al., 2015).

##### Procedures

For the first study, a literature review was previously conducted on designed instruments to assess performance in team sports in general and basketball in particular. The authors then defined all the items included in the first version of the tool. In the second stage of the study, the necessary criteria were established for being considered an expert. All the necessary documentation for the qualitative and quantitative assessment of the instrument was sent by email to 25 experts: a formal presentation of the study, the BALPAI and a template where they could make their assessments. Positive answers were received by email from the experts participating in the study. The experts were asked about: (i) the level of pertinence of the components of play actions (decision making, technical execution and final efficacy) and coding levels (inadequate action; neutral action; adequate action) to be evaluated; (ii) the level of comprehension of the components of play actions from the observational instrument; (iii) the need to include other play actions, or qualitative comments about play actions.

After the assessment of the experts, the criteria were defined for the modification, elimination or approval of the items according to the value obtained for Aiken’s (1985) V coefficient. The analysis of the internal consistency of the items was calculated with Cronbach’s α based on the values provided by the experts for the two content validity variables of adequacy and wording of each item.

#### Study 2: Assessment the Instrument Inter-Observer Reliability

##### Participants

For reliability purposes, youth players were recorded and assessed. The youth participants were attending the same state school class. A total of 25 fifth graders students (14 boys aged 10.78 years and 11 girls aged 10.85 years), from two different class groups (13 students from group A and 12 students from group B) from a school in the southwest of Spain took part in the study. Teachers, students and experts were informed of the study protocol, the participation of both groups and the research purposes. The students were informed that they would be filmed for later analysis. The basketball half court matches were part of the Physical Education classes, included in a basketball program of 15 sessions (55 min each) (González-Espinosa et al., 2017). The games were filmed in the last two sessions. The teams were created for the study and they did not have prior experience as a team. The teams were balanced by considering technical-tactical skills of all the students involved (Gracia et al., 2014). Teams were formed together by the teacher and the research staff.

##### Measures

Inter-observer reliability, or internal reliability, understood as the degree of agreement among the observers. In this case, the agreement among different observers concerning the description of several events is assessed (Thomas et al., 2015). In order to achieve high levels of reliability, all observers have received training in the use of instrument.

##### Procedures

Finally, the level of inter-observer reliability of the instrument was determined among three observers who used the BALPAI to record the play actions in a 3 × 3 basketball match. Only one hoop is used in the game and when a defense rebound occurs, the ball have to be returned outside the traditional three point-line before start attacking (Montgomery and Maloney, 2018). The three observers who intervened in this phase fulfilled all the previously defined inclusion criteria for being considered expert and, in addition, have time availability. For the observers to attain a minimum of reliability and objectivity in the codification, it was necessary to reach an agreement among them to permit an increase in the accuracy of the recordings of this human behavior (Medina and Delgado, 1999). The three observers received five training sessions (Muñoz et al., 2018).

The last two corresponded to the test of reliability among the observers for which each one recorded all the play actions in a filmed 3 × 3 basketball match. This game modality offers the players a greater opportunity to participate more successfully than in more numerous game modalities (Martínez-Fernández et al., 2015). Games were recorded with a SONY Full HD 1080 video camera at 60 fps; allowing experts to use slow motion and watch the videos as many times as they needed. The experts collected the data using an excel sheet designed for this purpose. This test assessed six subjects at the same time. Thus, the reliability analysis was made on the first 15 possessions in a match of 5 min duration on the part of each observer. The teams were established randomly, to avoid the polluting variable of the game level. The sample that participated in this study was composed of six students, three students per team. For this study, a game was selected in which only boys played, to avoid the contaminating variable of gender. The same clips were evaluated by the experts, who had no relation with the players nor were known to them. The experts were able to watch the video clips using Gamebreaker software (Sylvan Advantage, Hartford, Vermont) as many times as they thought fit, until they could make an adequate judgment. This option was determined, as they were continuous game actions, occurring simultaneously.

The parents of the players were informed about the study and gave their written consent in accordance with the Declaration of Helsinki. The study, with a full description of the protocols regarding recruitment and participation of the experts, was approved by the Ethics Committee of the University of Extremadura (no. 67/2017).

## Data Analysis

Firstly, content validity was calculated with Aiken’s V coefficient (Aiken, 1985). Its value goes from 0 to 1, with the latter marking perfect concordance among the experts with regard to the contents assessed. Aiken’s V coefficient score establishes which items should be eliminated, modified or retained. Aiken’s V was calculated following the algebraic equation modified by Penfield and Giacobbi (2004).

$$V=\frac{\bar{X}-l}{\text{k}}$$

Calculations were made using the free software program Visual Basic 6.0 (Merino and Livia, 2009), which makes it possible to obtain three factors: the range of valuations (maximum valuation - minimum valuation), Aiken’s V coefficient and the confidence intervals of 90, 95, and 99% using the score method (Penfield and Giacobbi, 2004).

The exact critical reference value for the acceptance of Aiken’s V was calculated using the initial formula proposed by Aiken (1985), applying the central limit theorem for large samples (m > 25). The number of experts was 13 (n), the number of items 66 (m), with an answer range of 10 (c); applying the value of the constant of content validity of 95 and 99% (z).

$$\frac{z}{0.2\sqrt{\frac{3\mathrm{mn}(c-1)}{\left(c+1\right)}}}+0.5$$

The confidence level of 95% was considered to obtain the exact critical value for an item to be included and a value of 0.68 was attained. Similarly, the confidence level of 99% was considered to obtain the cut-off point for the modification of the tasks attaining a value of 0.75. Table 2 shows the criteria used for the acceptance, modification or elimination of the items from the instrument.

**Table 2:** Criteria for the acceptance, modification or elimination of the items.

	Wording			
		>0.75	[0.68–0.75]	<0.68
**Adequacy**	>0.75	Correct	Wording is modified	Wording is modified
	[0.68–0.75]	Adequacy is modified	Adequacy and Wording are modified	Adequacy and Wording are modified
	<0.68	Eliminated	Eliminated	Eliminated

Cronbach’s α was then used for the analysis of internal consistency. This coefficient presents values between 0 and 1 and shows the reliability of the studied instrument. A value of 1 is perfect reliability but >0.70 is considered valid (Field, 2009). SPSS 21.0 software was used to analyze the internal consistency of the instrument (IBM SPSS Statistics for Windows. Armonk, NY: IBM Corp.).

Finally, the inter-rater agreement of the instrument was studied. As three raters intervened in the reliability analysis and the number of cases which had to be distributed in each of the categories of the instrument was unknown, it was necessary to use the *Free-Marginal Multirater Kappa* (*Multirater* κ_free_) (Randolph, 2005). The computer application Online Kappa Calculator^1^ was used for the interobserver reliability analysis. The variables analyzed were categorical. The following values were used to interpret the strength value of the Multirater κ_free_: (i) a value of 0.00 or less was considered poor agreement; (ii) a value of 0.00 to 0.20 slight agreement; (iii) a value of 0.21 to 0.40 fair agreement; (iv) a value of 0.41 to 0.60 moderate agreement; (v) a value of 0.61 to 0.80 substantial agreement; and (vi) a value of 0.81 to 1 was considered almost perfect agreement (Landis and Koch, 1977; Altman, 1991).

## Results

Table 3 shows the mean values obtained for each of the items in the BALPAI instrument as well as the value of Aiken’s V coefficient. These high values suggest a high content validity in our results.

**Table 3:** Results of Aiken’s V coefficient for the 11 variables of the BALPAI in each of the play action components.

	Dribbling						Shooting						Passing					
	DM^∗^		TE		FE		DM		TE^∗^		FE		DM^∗^		TE		FE	
	A	W	A	W	A	W	A	W	A	W	A	W	A	W	A	W	A	W
M	7.57	7.50	8.50	8.14	8.57	8.71	9.00	8.29	8.50	7.36	8.07	9.07	8.14	7.57	8.29	8.07	9.15	9.50
V	0.79	0.72	0.83	0.79	0.84	0.86	0.89	0.81	0.83	0.71	0.79	0.90	0.79	0.73	0.81	0.79	0.91	0.94

	**Receiving**						**Passing and playing**						**Occupying free spaces**					
	**DM^∗^**		**TE**		**FE**		**DM**		**TE^∗^**		**FE**		**DM^∗^**		**TE**		**FE**	
	**A**	**W**	**A**	**W**	**A**	**W**	**A**	**W**	**A**	**W**	**A**	**W**	**A**	**W**	**A**	**W**	**A**	**W**

M	8.86	8.14	8.86	8.29	8.57	9.50	8.08	8.08	9.07	8.50	9.29	8.50	9.07	8.21	8.93	8.64	8.79	8.71
V	0.87	0.79	0.87	0.81	0.84	0.94	0.79	0.79	0.90	0.83	0.90	0.83	0.90	0.80	0.88	0.85	0.87	0.86

	**Offensive rebound**						**Defensive rebound**						**On ball defense**					
	**DM^∗^**		**TE**		**FE**		**DM**		**TE^∗^**		**FE**		DM^∗^		**TE**		**FE**	
	**A**	**W**	**A**	**W**	**A**	**W**	**A**	**W**	**A**	**W**	**A**	**W**	**A**	**W**	**A**	**W**	**A**	**W**

M	9.21	8.86	8.93	9.14	8.86	8.71	9.36	9.21	9.21	8.79	9.36	9.07	9.29	8.93	9.36	8.50	9.00	9.00
V	0.91	0.87	0.88	0.90	0.87	0.86	0.93	0.91	0.91	0.87	0.93	0.90	0.92	0.88	0.93	0.83	0.89	0.89

	**Off ball defense**									**Assisting and recovery/defensive change**								
	**DM^∗^**			**TE**			**FE**			**DM**			**TE^∗^**			**FE**	
M	9.21	8.21		8.93	8.64		9.21	8.79		8.57	8.21		8.50	8.14		9.14	8.65	
V	0.91	0.80		0.88	0.85		0.91	0.87		0.84	0.80		0.83	0.79		0.90	0.85	

*M, Arithmetic mean; V, Aiken’s V; A, Adequacy; W, Wording; DM, Decision making; TE, Technical execution; FE, Final efficacy. ^∗^ = Item where W was modified.*

The values obtained indicate that it was not necessary to eliminate any of the items according to the criteria established in the literature. A very demanding criterion was established for the elimination or modification of the items. However, there was no need to make any changes in Adequacy (A). Changes only had to be made in the Wording (W) of the following items: DM in dribbling; TE in shooting; DM in passing. The contributions that the group of experts made in their subjective valuations were used as a reference to carry out the necessary modifications. These modifications were made in all the items suggested with the aim of improving the instrument, despite not being necessary in some items. The instrument was sent back to the experts, who accepted the final version.

All the variables in the instrument attained a value for Cronbach’s α of greater than 0.90 except Decision Making (0.87) (Table 4). The results of the internal consistency and IO reliability tests indicated high levels of reliability for this instrument. The analysis confirmed the high level of internal consistency.

**Table 4:** Results of the analysis of the internal consistency of the instrument.

	Adequacy	Wording	Offense	Defense	DM	TE	FE	Instrument
α	0.959	0.950	0.953	0.933	0.876	0.917	0.921	0.969
N	33	33	42	24	22	22	22	66

*DM, Decision making; TE, Technical execution; FE, Final efficacy; N, Number of items.*

Finally, Table 5 shows the results regarding inter-observer reliability where all the items attained a value of above 0.81 and some equal to 1.

**Table 5:** Results of Interobserver reliability for each of the 11 variables in the BALPAI in each of the components of the play actions.

	Dribbling			Shooting			Passing			Receiving			Passing and playing			Occupying free spaces		
	DM	TE	FE	DM	TE	FE	DM	TE	FE	DM	TE	FE	DM	TE	FE	DM	TE	FE
Po	0.94	0.94	0.89	0.93	0.99	0.99	0.94	0.99	0.99	1	0.94	1	1	1	1	0.94	1	0.92
κ_free_	0.92	0.92	0.84	0.90	0.99	0.99	0.92	0.99	0.99	1	0.91	1	1	1	1	0.91	1	0.88

	**Offensive rebound**			**Defensive rebound**			**On Ball defense**			**Off ball defense**			**Assisting and recovery/defensive change**					
	DM	TE	FE	DM	TE	FE	DM	TE	FE	DM	TE	FE	DM		TE		FE	

Po	0.97	1	1	0.97	0.93	1	0.93	0.96	0.96	1	1	0.98	0.99		0.99		0.99	
κ_free_	0.96	1	1	0.96	0.90	1	0.90	0.95	0.95	1	1	0.97	0.99		0.99		0.99	

*Po, Percentage of global agreement; κ_free_, Free-marginal kappa; DM, Decision making; TE, Technical execution; FE, Final efficacy; N, Number of items.*

## Discussion

The purpose of the present study was to design an instrument for the specific and general assessment of basketball play. It had to assess offensive and defensive play actions, with and without the ball, and their three components. The results show that the BALPAI is the most complete of the existing instruments and has a high level of content validity, internal reliability and inter-observer reliability.

To validate the instrument, it was necessary to have expert opinion on its application (García-Martín et al., 2016). In the case of studies involving the judgment of experts a series of recommendations have to be taken into account, like those mentioned by Bulger and Housner (2007), Dunn et al. (1999), Escobar-Pérez and Cuervo-Martínez (2008) and Skjong and Wentworth (2001): the quality of the inclusion criteria, the number of experts necessary for this type of study, the preparation of the instructions and assessment templates, the procedure for collecting the quantitative and qualitative statistics as well as the adequate statistical analysis to give the instrument validity and reliability.

With regard to the sample of experts used in the investigation, several studies have established the range between two and twenty (Rubio et al., 2003), other researchers consider that ten is a reliable number (Hyrkäs et al., 2003), or three minimum, five acceptable, and ten, the ideal number (Lynn, 1986). In this study the number of experts who participated by offering their assessment of all the items in the instrument was 13, corresponding to 53% of the initially detected population according to demanding inclusion criteria, and fulfilling the requisites established in the literature.

The qualitative assessments of the experts are equally important when developing and perfecting the items of the instrument (Bulger and Housner, 2007; Carretero-Dios and Pérez, 2007; Padilla et al., 2007), and in this case, a deficiency was revealed in the quantification of the values of the questionnaire in some items. The experts’ contributions were directed at improving the wording, clarifying the expressions so that they did not generate doubts in the future codifiers. The value 2 did not correctly discriminate between the values one and three of the instrument. Thus, according to the suggestions of several experts, the value 2 was re-worded so that the difference with the other values was even clearer. Furthermore, although not as clearly as in the previous assessments, the experts indicated that the item on the technical execution of the shot led to misunderstandings in the way it was expressed. It was suggested that the description be modified, especially with regard to the part referring to “the starting point for the shot.” Many of the assessments of the experts were reflections on the instrument which, in some cases, made it possible to define its items more clearly and accurately (Wiersma, 2001).

The content validity showed values in all the items of over 0.70 for Aiken’s V, so that it was only necessary to modify the wording of three of the 66 items. The demands of inclusion, modification and exclusion criteria were increased, 95% confidence criterion was established for acceptance or elimination of an item, and 99% for its modification (Penfield and Giacobbi, 2004). Previous studies have had lower levels of demand (Ortega et al., 2008; García-Martín et al., 2016; García-Santos and Ibáñez, 2016). These items were reworded as in previous studies (Bulger and Housner, 2007; Ortega et al., 2008; Villarejo et al., 2014). When the internal consistency of the instrument was analyzed, it was seen that other tools (questionnaires, interviews, instruments...) that had already been published and validated, present lower values than those attained by the BALPAI, overall Cronbach’s α = 0.97 vs. Cronbach’s α = 0.72 of IOVAB for basketball referees (García-Santos and Ibáñez, 2016); vs. Cronbach’s α = 0.94 of Socio-emotional questionnaire (Gómez-Carmona et al., 2014); and vs. Cronbach’s α = 0.96 of programs for sports education in the school context (Gonzalez-Espinosa et al., 2017). The values for inter-observer reliability were over 0.84 in Kappa coefficient thus being perfect or nearly perfect (Altman, 1991; Landis and Koch, 1977). The BALPAI tool has demonstrated very good inter-observer reliability in its practical application, with values of the Kappa coefficient between 0.84 and 1 in the 11 variables and the three components of the play actions (decision making, technical execution and efficacy), considered as an almost perfect agreement (Landis and Koch, 1977; Altman, 1991). Painczyk et al. (2018) used Cohen’s Kappa coefficient to determine the interobserver reliability of a match evaluation notational system in Rugby Union, with values lower than those found in this study. These results confirm the quality of the design of the tool, since the observers who have used it have shown great concordance evaluating game actions. All the analyses carried out confirmed the validity and reliability of the designed instrument.

Differences between instruments have been pointed out, but possible explanation for these differences have to be exposed. Painczyk et al. (2018) analyzed seven complete rugby union games, observing several performance indicators with different operational definitions. BALPAI variables and operational definitions used for reliability purposes in our study were smaller. In addition, BALPAI was design for make easier observations, in the number of variables analyzed as in player’s skill level. Moreover, main concerns of reliability studies are the clear operational definitions of each variable (Painczyk et al., 2018; O’Donoghue, 2007) and the observers training processes (Liu et al., 2017). Researchers that carried out the BALPAI reliability analysis have participated in the development and validation of the instrument, showing a great understanding of variables and definitions. Moreover, these researchers have been defined as experts, that achieve better reliability values that inexperienced ones (Painczyk et al., 2018).

The importance of observational tools has been previously reported in other sports and contexts (Llobet-Martí et al., 2016). Other testing procedures have reported different approaches such as creativity or divergent thinking (Memmert, 2010). These approaches will have to be taken into consideration in future research. In addition, further research should also suggest the analysis of coaches in order to improve the learning process with the validation of observational tools (Nicholls and Worsfold, 2016).

## Limitations

Basketball Learning and Performance Assessment Instrument contains many items and components, thus the assessment process can be quite hard. This process can be focused on a single item at a time, or two or several. As well as helping students to pay attention to important information that they should learn, it simplifies the assessment process for teachers and coaches. Moreover, before using the BALPAI, teachers and coaches must undergo a training period in how to implement it, leading to a better use and recognition of play behaviors that can be quite subjective.

## Conclusion

The BALPAI, has shown, during this first phase of validation, to be a valid and reliable instrument for assessing learning in basketball in PE classes, and has proved to be more complete than previously published tools, on which its design was based. It also possesses a high level of reliability in the codification of the play actions.

Teachers can use BALPAI in their teaching programs in Physical Education as part of the evaluation. The use of this tool will make it possible to assess the progress of players in the educational context, assessing students’ learning during the school year. In addition, teacher can assess different teaching programs, comparing studentsŕesults in both programs. The repetition of the assessment of students will make it possible to confirm if the intervention programs used in their training are effective.

## Ethics Statement

This study have been approved by the Ethics Committee of the University of Extremadura.

## Author Contributions

SI: conceptualization, data collection, formal analysis, investigation, methodology, writing review, and editing. SM-F: data collection, formal analysis, investigation, writing original draft. SG-E: data collection, formal analysis and investigation. SF: supervision, writing review, and editing. JG-R: supervision, writing original draft, writing review, and editing.

## Conflict of Interest Statement

The authors declare that the research was conducted in the absence of any commercial or financial relationships that could be construed as a potential conflict of interest.

## Footnotes

1 http://justusrandolph.net/kappa/
