# Supplementary material for: Designing and Validating a Basketball Learning and Performance Assessment Instrument (BALPAI)
Source: Front Psychol. 2019 Jul 10;10:1595. doi: 10.3389/fpsyg.2019.01595 (PMC6635796; doi:10.3389/fpsyg.2019.01595)
Supplement: Supplementary file 1 [file Data_Sheet_1.PDF]

Annex 1

Play action	Component	Assessment
1. Dribbling	Decision making	1. Dribbling without tactical intentionality*<br>2. Dribbling keeping the ball, but without tactical intentionality*<br>3. Dribbling with tactical intentionality*<br>*Tactical intentionality in dribbling: dribbling which serves to improve the shot or the possibility of passing retaining possession
	Technical execution	1. Dribbling the ball with the hand nearest to the opponent and looking at the ball or the floor<br>2. Dribbling the ball with the hand nearest the opponent or looking at the ball or the floor<br>3. Dribbling the ball with the hand farthest from the opponent and looking at the game
	Final efficacy	1. Loss of possession of the ball and/or committing a foul<br>2. Maintaining possession of the ball without generating an advantage *<br>3. Generating an advantage* with the bounce<br>*Generating advantage with the bounce means that thanks to the bounce it was possible to pass or shoot
2. Shooting	Decision making	1. Making a shot when there is clear defensive pressure and there is a team mate in a more favourable condition to shoot<br>2. Making a shot when there is clear defensive pressure or there is a team mate in a more favourable condition to shoot<br>3. Making a shot when there is no clear defensive pressure and when there is a more favourable condition to shoot than that of the team mates
	Technical execution	1. The shot is not made in a continuous kinetic chain and the hand does not end up pointing at the hoop<br>2. The shot is made without a continuous kinetic chain or the hand does not end up pointing at the hoop<br>3. The shot is made in a continuous kinetic chain and the hand ends up pointing at the hoop
	Final efficacy	1. The shot is blocked by a defender and/or does not touch the hoop or backboard<br>2. The shot does not pass through the hoop, but touches the hoop or the backboard<br>3. The shot goes through the hoop
3. Passing	Decision making	1. Passing to a team mate with high defensive pressure and also when having the opportunity to shoot or advance to the basket<br>2. Passing to a team mate with high defensive pressure or when having the opportunity to shoot or advance to the basket<br>3. Passing to a team mate without high defensive pressure and when not having the opportunity to shoot or advance to the basket
	Technical execution	1. The pass does not start from a maximally functional zone* far from the direct defender or its final destination is not where the receiver is asking for it<br>2. The pass starts from a maximally functional zone* far from the direct defender or its final destination is where the receiver is asking for it<br>3. The pass starts from a maximally functional zone* far from the direct defender and its final destination is where the receiver is asking for it

		* A maximally functional zone is one that allows the most possible functions with the ball (zone generally between the shoulders and the hips) and is suitable for the players' level and the technical gesture of the pass
	Final efficacy	1. The pass does not reach the intended receiver or is intercepted by the defence<br>2. The pass reaches the intended receiver, but does not allow immediately carrying out the play action<br>3. The pass reaches the intended receiver and allows immediately carrying out the play action
4. Receiving	Decision making	1. The ball is received without generating uncertainty in the direct opponent and not in the direction of the basket<br>2. The ball is received generating uncertainty in the direct opponent or in the direction of the basket<br>3. The ball is received generating uncertainty in the direct opponent and in the direction of the basket
	Technical execution	1. The ball is received without securing* it and without placing it in a maximally functional zone**<br>2. The ball is received and secured* or placed in a maximally functional zone**<br>3. The ball is received and secured* and placed in a maximally functional zone**<br>* Securing the ball is holding the ball securely so that possession is not lost, normally using both hands.<br>** A maximally functional zone is one that allows the most possible functions with the ball (zone generally between the shoulders and the hips) and is suitable for the players' level and the technical gesture of the pass.
	Final efficacy	1. It is not possible to control the ball<br>2. The ball is controlled, but there is no immediate possibility of carrying out another play action<br>3. The ball is controlled and there is an immediate possibility of carrying out another play action
5. Passing Play	Decision making	1. There is no play action* after making the pass<br>2. After making the pass player plays* through an unsuitable space**<br>3. After making the pass player plays* through a suitable space**<br>*The concept of playing after a pass is considered as making a movement to favour the flow of the game, giving an option for a pass to the player with the ball or freeing the space occupied to be occupied by another player. After the pass the player can progress (cut) to the basket, increase (clear) the space or support the player with the ball.<br>**A suitable space for playing after passing is one that does not hinder the action of the player with the ball.
	Technical execution	1. Playing without looking at the team mate with the ball and without showing hand(s) for receiving<br>2. Playing looking at the team mate with the ball or showing hand(s) for receiving<br>3. Playing looking at the team mate with the ball and showing hand(s) for receiving
	Final efficacy	1. Play does not permit freeing oneself from the marking of the direct defender and does not give the possibility of receiving a pass<br>2. Play permits freeing oneself from the marking of the direct defender or gives the possibility of receiving a pass<br>3. Play permits freeing oneself from the marking of the direct defender and gives the possibility of receiving a pass

6. Occupying free spaces without the ball	Decision making	1. The initial distance to the player with the ball is not adequate* (very near or very far) and is not adjusted when the player with the ball changes position<br>2. The initial distance to the player with the ball is adequate* or is adjusted when the player with the ball changes position<br>3. The initial distance to the player with the ball is adequate* and is adjusted when the player with the ball changes position<br>* An adequate distance is one that makes it possible to make a pass and does not hinder the action of the player with the ball.
	Technical execution	1. Player does not maintain a double threat posture* nor a triple threat position**<br>2. Player maintains a double threat posture* or a triple threat position**<br>3. Player maintains a double threat posture* and a triple threat position**<br>* A double threat posture is one that permits making an immediate bounce or pass on receiving the ball requiring a semi-flexed posture and hands ready to receive.<br>** A triple threat position is a location from which an immediate shot can be performed on receiving the ball. It should be suitable for the players' level.
	Final efficacy	1. The player without the ball hinders the action of the player with the ball<br>2. The player without the ball does not hinder the action of the player with the ball, but does not allow a pass to be made<br>3. The player without the ball does not hinder the action of the player with the ball, and allows a pass to be made
7. Offensive rebound	Decision making	1. When the shot is made player makes no move to get the offensive rebound<br>2. When the shot is made player goes to get the offensive rebound, but does not go through a suitable space to do so*<br>3. When the shot is made player goes to get the offensive rebound, and goes through a suitable space to do so*<br>* A suitable move is one that permits player to approach the basket in a straight line when there are no defenders in the way or if there are it is considered a suitable move when player goes through an unseen space for the defender.
	Technical execution	1. Player does not make a move to get the rebound<br>2. Player makes a move adapted to the time and space for getting the rebound, but without using arms correctly<br>3. Player makes a move adapted to the time and space for getting the rebound, using arms correctly
	Final efficacy	1. Player makes no move to get the offensive rebound<br>2. Player does not force defence to take any action to secure defensive rebound<br>3. Player manages to catch offensive rebound or forces defence to secure defensive rebound
8. Defensive rebound	Decision making	1. When the shot is made player makes no move to get the defensive rebound<br>2. When the shot is made player takes action to get the defensive rebound without blocking the rebound of the nearest opponent<br>3. When the shot is made player looks for nearest opponent to block the rebound

	Technical execution	1. Player does not contact nearest opponent to block the rebound<br>2. Player contacts nearest opponent, but does not turn the body to block the rebound with the back (provided there is the necessary time to do so)<br>3. Player contacts nearest opponent and turns the body to block the rebound with the back (provided there is the necessary time to do so)
	Final efficacy	1. Player does not contact nearest opponent<br>2. Player contacts nearest opponent, but does not hinder/hamper/obstruct the action to get the offensive rebound<br>3. Player gets defensive rebound or contacts nearest opponent and hinders/hampers/obstructs the action to get the offensive rebound.
9. On ball marking	Decision making	1. Player does not get between the offensive player and the basket and is at an unsuitable distance (very near or very far)<br>2. Player gets between the offensive player and the basket or is at a suitable distance<br>3. Player gets between the offensive player and the basket and is at a suitable distance
	Technical execution	1. Player does not take up a basic defensive posture and does not use the hands to hinder/hamper/obstruct the offensive player's action adjusting to his movement<br>2. Player takes up a basic defensive posture or uses the hands adequately to hinder/hamper/obstruct the offensive player's action adjusting to his movement<br>3. Player flexes his lower body and uses the hands adequately to hinder/hamper/obstruct the offensive player's action adjusting to his movement<br>* A basic defensive posture is one that makes it possible to react and move quickly to the movement of the direct opponent (with a semiflexion of the lower body).
	Final efficacy	1. Player does not hinder/hamper/obstruct the action of the player with the ball<br>2. Player hinders/hampers/obstructs the action of the player with the ball<br>3. Player recovers possession of the ball or considerably hinders/hampers/obstructs the action of the player with the ball
10. Off ball marking	Decision making	1. Player does not modify location according to the defensive triangle* and does not adequately modify the position according to the situation of the direct opponent or the player with the ball (on the weak side or the strong side)<br>2. Player modifies location according to the defensive triangle* or adequately modifies the position according to the situation of the direct opponent or the player with the ball (on the weak side or the strong side<br>3. Player modifies location according to the defensive triangle* and adequately modifies the position according to the situation of the direct opponent or the player with the ball (on the weak side or the strong side<br>*The defensive triangle is composed of the hoop, the direct opponent and the opponent with the ball.

	Technical execution	1. Player does not maintain a basic defensive posture* and does not keep both the player with the ball and the direct opponent in field of vision<br>2. Player maintains a basic defensive posture* or keeps both the player with the ball and the direct opponent in field of vision<br>3. Player maintains a basic defensive posture* and keeps both the player with the ball and the direct opponent in field of vision<br>*** A basic defensive posture is one that makes it possible to react and move quickly to the movement of the direct opponent (with a semiflexion of the lower body).**
	Final efficacy	1. Player does not hinder/hamper/obstruct reception of the ball<br>2. Player hinders/hampers/obstructs reception of the ball<br>3. Player prevents reception of the ball or considerably hinders/hampers/obstructs it
11. Defensive help/defensive change	Decision making	1. When there is penetration to the basket there is no defensive help on the part of the off-ball attacker's defender<br>2. When there is penetration to the basket there is defensive help on the part of the off-ball attacker's defender but not an adequate subsequent action*<br>3. When there is penetration to the basket there is defensive help and an adequate subsequent action on the part of the off-ball attacker's defender but not an adequate subsequent action*<br>*The subsequent action to defensive help depends on how the team mate that has been passed acts. If the latter manages to recover the direct opponent, the defender that gave the help should carry out a defensive recovery, while if the team mate that was passed does not recover the direct opponent the adequate subsequent action is a defensive change.
	Technical execution	1. Defensive help is not given with the body flexed or extended (according to whether the defensive help is from a defender in the first passing line or not) and player does not use it to totally shut down the trajectory of the player with the ball to the basket<br>2. Defensive help is given with the body flexed or extended (according to whether the defensive help is from a defender in the first passing line or not) or player uses it to totally shut down the trajectory of the player with the ball to the basket<br>3. Defensive help is given with the body flexed or extended (according to whether the defensive help is from a defender in the first passing line or not) and player uses it to totally shut down the trajectory of the player with the ball to the basket
	Final efficacy	1. The penetration of the offensive player with the ball is not slowed nor does player hinder/hamper/obstruct a shot at the hoop under good conditions<br>2. The penetration of the offensive player with the ball is slowed or player hinders/hampers/obstructs a shot at the hoop under good conditions<br>3. The penetration of the offensive player with the ball is slowed and player considerably hinders/hampers/obstructs a shot at the hoop under good conditions
